# Editorial: Community series in the wildlife gut microbiome and its implication for conservation biology, volume II

**DOI:** 10.3389/fmicb.2023.1329928

**Published:** 2023-12-20

**Authors:** Lifeng Zhu, Jianjun Wang

**Affiliations:** ^1^School of Medicine and Holistic Integrative Medicine, Nanjing University of Chinese Medicine, Nanjing, China; ^2^State Key Laboratory of Lake Science and Environment, Nanjing Institute of Geography and Limnology, Chinese Academy of Sciences, Nanjing, China

**Keywords:** gut microbiome, reintroduction, anthropogenic pressure, conservation, function

Over the past decade, mounting evidence has highlighted the pivotal role of the gut microbiome in the physiological development, nutrient absorption, and immune health of wildlife (Wei et al., 2019). The integration and application of non-invasive sampling methods, genomics, and metagenomics has provided valuable insights into important aspects such as population status, genetic diversity, and health conditions of wild animal species (Wei et al., 2019; Huang et al., 2021). These approaches offer invaluable perspectives and tools for investigating the connections between the gut microbiome and wildlife health, while concurrently enhancing our understanding of the ramifications of human-induced disruptions, habitat alterations, and environmental pollution on the host symbiotic microbiome (Menke et al., 2017; Lavrinienko et al., 2018; Degregori et al., 2021). In addition, employing comprehensive research methods that delve deeply into the role and influencing factors of the gut microbiota enable a more comprehensive understanding and protection of the health and habitat of wildlife, facilitating effective monitoring and improvement of conservation efforts for endangered wildlife (Yao et al., 2019; Zhu et al., 2021a). Moreover, by elucidating the correlation between gut microbiota and conservation, it becomes feasible to further assess the efficacy of conservation strategies and furnish scientific substantiation for the formulation of adaptive management strategies (Wei et al., 2019; Huang et al., 2023).

Building on Volume I (Zhu et al., 2021b), the second volume of the Research Topic titled *Community Series in the Wildlife Gut Microbiome and Its Implication for Conservation Biology* in *Frontiers in Microbiology* comprises a comprehensive collection of 20 original research articles that provide valuable insights for further study of the relationship between wildlife gut microbes and conservation biology. Below is a summary of the articles included in the volume II of this topic.

## The specificity of gut microbiome in wildlife in response to varying conservation conditions

Over the years, captive breeding, translocation, and reintroduction have proven to be effective strategies in safeguarding endangered wildlife, rendering substantial contributions to the realm of wildlife conservation endeavors (Harding et al., 2016; Gross et al., 2023; Smith et al., 2023). Nonetheless, as summarized in our editorial in the first volume at 2021, the environmental changes resulting from different conservation strategies pose challenges to the ecological adaptability of the host species (Zhu et al., 2021b). In this regard, the gut microbiome exhibits specificity as a potential indicator reflecting the health status and adaptability of wild animals (de Jonge et al.; Zhou et al., 2022). In the second volume, we have made efforts to acquire additional relevant articles, which further expand our understanding of the specific gut microbiota responses in different wild animal species under variable conservation strategies. On one hand, in comparison with wild populations, captive conservation can exert a beneficial influence on the gut microbiota structures and functions of wild animals. In this topic, it has been showed that captive alpine musk deer (*Moschus chrysogaster*) individuals exhibit higher alpha diversity (alpha and beta diversity) and stronger functions of chemoheterotrophy and fermentation, which could potentially enhance the stability and complexity of the gut microbial community (Zhang et al.). This reinforcement contributes to the host's ecological adaptation mechanisms in the habitat environment. On the other hand, captive breeding may have detrimental effects on the host's health by increasing the abundance of potential pathogenic bacteria in the gut. For instance, Tang et al. discovered a higher presence of potentially pathogenic bacteria in the gut microbiota of captive tokay gecko (*Gekko gecko*). Similarly, Xia et al. ascertained reduced α-diversity, heightened abundance of potential pathogenic bacteria (e.g. *Streptococcus* and *Sarcina*), and the enrichment of antibiotic resistance (KEGG pathway) of gut microbiome within captive populations of tibetan macaques (*Macaca thibetana*). In addition, in a successful translocation case involving koalas (*Phascolarctos cinereus*), Blyton et al. found that the formation of the koala gut microbiota is primarily determined by the acquisition and development of microbial communities during early life stages, while dietary variations resulting from the translocation process did not significantly affect the host gut microbiota. In summary the gut microbiota of different wildlife species exhibits remarkable specificity in their responsiveness to alterations in the habitat environment. Consequently, fully elucidating the host gut microbiota's reaction under varying conservation conditions assumes pivotal practical significance for safeguarding and managing endangered wildlife.

## The negative response of wildlife gut microbiome to the ecological pressures of the Anthropocene

In the Anthropocene epoch, the escalation of human activities, encompassing urbanization, agricultural expansion, hunting, and logging, etc., has wrought severe devastation upon global biodiversity and the pristine habitats of wildlife (Hockings et al., 2015; Otto, 2018; Schmidt et al., 2020). Wildlife encounters grave perils arising from habitat fragmentation and numerous anthropogenic disturbances, constituting one of the pivotal concerns impinging upon wildlife conservation endeavors (Sánchez-Barreiro et al., 2021). These disturbances engender not only direct biodiversity loss but also exert deleterious ramifications on wildlife well-being through modifications to the symbiotic microbiome, concomitantly amplifying the risk of localized extinction on a population scale (Barelli et al., 2015; Hockings et al., 2015; Xi et al., 2023). Hence, delving into the response of wildlife gut microbiome to the ecological stressors imposed by human interference furnishes indispensable insights for the pursuit of animal conservation initiatives (Cortés-Avizanda et al., 2022). In this topic, it has been reported that the effects of the human environmental factors on the host microbiome. For example, Wasimuddin et al. studied the gut microbiome of gray-brown mouse lemurs (*Microcebus griseorufus*) in south-western Madagascar. They revealed the impact of anthropogenic disturbance on the gut microbiome of wild lemurs, highlighting its disruptive nature and negative health consequences, with a decline in gut microbial diversity and beneficial bacterial species, accompanied by a slight rise in potentially pathogenic bacteria (Wasimuddin et al.). Similarly, Zhou et al. assessed the impact of urbanization on the symbiotic microbial community of wild amphibians (*Pelophylax nigromaculatus, Fejervarya multistriata*, and *Bufo gargarizans*) and found that although urbanization increased the diversity of symbiotic microbiota and the number of keystone species taxa, it came at the cost of a depletion of the stability and complexity of the microbial community.

## Spatiotemporal dynamics of environmental variables changes affecting the host-microbiome

The macroscopic spatiotemporal dynamics of environmental variables exert a substantial influence on the structure, function, and adaptive evolution of the host-microbiome (Ma et al., 2019; Li et al., 2023). The changes in dietary resources due to seasonal fluctuations can induce alterations in the community structure of the host microbiome, thereby significantly impacting the physiological functions of the host (Huang et al., 2022; Li et al., 2023). The issue was investigated by Qin et al. and Li et al. in this topic. It was found by Qin et al. that seasonal dietary changes significantly impact the gut microbiota diversity (α and β diversity) and metabolic function of goitered gazelles (*Gazella subgutturosa*). Specifically, during winter, both the alpha diversity and metabolic function of gut microbiome were higher compared to summer. Li et al. revealed that seasonal changes in the gut microbiota of golden snub-nosed monkeys (*Rhinopithecus roxellana*) are primarily caused by variations in macronutrient intake, and the metabolic adaptation of gut microbiota helps the host compensate for inadequate macronutrient intake. Furthermore, at global geographic scales, gradient variation, including altitude and latitude, may also influence the functional adaptation and convergent evolution of the host-microbiome through stringent ecological selection pressures (Karl et al., 2018; Ma et al., 2019; Zuo et al., 2022). Wang et al. studied the adaptation of ungulates gut microbiome to the environmental extremes of the Tibetan Plateau, and their results showed that while high altitude may not surpass the phylogenetic factors in driving the convergent evolution of ungulate gut microbiome compositions, it does play a significant role in in promoting the convergent evolution of alpha diversity and indicator microbiota within the gut microbiome of ungulates. Another study presented a latitudinal pattern of gut microbial diversity in frogs (*Fejervarya limnocharis*) along the eastern coast of mainland China, showing a significant negative correlation between alpha diversity and the latitudinal gradient (Zhao et al.).

## Conclusion and perspectives

Building upon the investigations of the gut microbiome structure and functional adaptations in wild animals explored in Volume I (Zhu et al., 2021b), we further delve into examining the link between their gut microbiome and conservation biology within this topic. Overall, the current efforts in this field contribute to the reconstruction and maintenance of healthy microbial ecosystems in wildlife, enabling effective responses to external disturbances and threats originating from human or natural environments. However, as emphasized in our previous editorial in Volume I regarding the significance of sequencing depth, bioinformatics analysis, and database quality in host microbiome research, there will be an increased focus on whole-genome and multi-omics technologies (Wei et al., 2019; Skarzyńska et al., 2020; Zhu et al., 2021b; Li et al., 2022). In this topic, Zhu et al. employed a combination of metabolomics and 16S rDNA sequencing techniques to elucidate the association of host metabolism and gut microbiome with phylogeny and environmental adaptation in mountain dragons. This further highlights the value and potential of multi-omics technologies in future research, providing new insights into the study and conservation of the gut microbiome in wildlife. The theoretical research on gut microbiome has provided important guidance for the conservation and management of endangered animals. Gut microbiome of different wild animals showed specific responses to environmental changes (e.g. captivity or translocation), which subsequently influence the ecological adaptability of their hosts (Tang et al.; Xia et al.; Zhang et al.). Furthermore, gut microbiome holds the potential to serve as biomarkers for captive or reintroduced hosts at different stages of life, thus enabling more effective monitoring and management of the health status of endangered wildlife (Huang et al., 2023; Zhou et al.). Simultaneously, this has also fostered the development of new approaches for assessing the adaptive capacity and survival status of endangered animals, providing a basis for implementing appropriate conservation measures and management strategies. Undoubtedly, future research will continue to expand our understanding of the role and value of gut microbiota in endangered wildlife, making significant contributions to the conservation of biodiversity and ecosystem health.

## Author contributions

LZ: Writing – original draft, Writing – review & editing. JW: Writing – original draft.
